# Hypokalemia Masquerading as Large Vessel Occlusion Stroke: A Case Report

**DOI:** 10.7759/cureus.94179

**Published:** 2025-10-09

**Authors:** Philip S Nawrocki, Brent T Rau

**Affiliations:** 1 Department of Emergency Medicine, Allegheny Health Network, Pittsburgh, USA

**Keywords:** air ambulance, case report, emergency medical services, hypokalemia, stroke

## Abstract

This report describes a case of hypokalemia-induced hemiplegia and altered mental status masquerading as an acute large vessel occlusion (LVO) stroke, and provides a brief discussion of stroke mimics. An air ambulance was dispatched to rendezvous with a ground emergency medical services (EMS) crew to manage a 75-year-old female patient with altered mental status and left-sided weakness. The patient was transported to a comprehensive stroke center (CSC), where neuroimaging failed to demonstrate evidence for acute stroke, although the patient was found to have severe hypokalemia. The patient’s symptoms rapidly resolved following electrolyte repletion. This report highlights that prehospital and emergency department clinicians should have a familiarity with and a high index of suspicion for common stroke mimics.

## Introduction

Acute stroke is the fifth leading cause of death in the United States [1], with an estimated 795,000 strokes reported in the country annually [2]. Emergency departments play a crucial role in the early advanced diagnostics and treatment for acute stroke, although prehospital care systems have an equally important role in stroke recognition, appropriate triage to stroke centers, and early management. Prehospital guidelines emphasize public outreach and education, the use of a stroke assessment tool, hospital prearrival notification, and accurate triage to centers capable of intravenous thrombolytic administration [3]. Stroke mimics involve clinical presentations that appear similar to ischemic or hemorrhagic strokes; these have been reported to occur in 24.8-38% of acute presentations of neurologic deficits [4-6]. The limited resources and diagnostic tools available in the prehospital setting limit the ability of the prehospital provider to assess for and detect mimics rather than actual strokes. This report describes the case of a patient with severe electrolyte derangements masquerading as an acute large vessel occlusion (LVO) stroke.

## Case presentation

A ground emergency medical services (EMS) Advanced Life Support crew was dispatched to a private residence for an unresponsive 75-year-old female. Upon the arrival of the first responder, the patient was noted to be minimally responsive only to painful stimulation but was breathing spontaneously, and had a point-of-care glucose of 254 mg/dL. The first responding paramedic assessed the patient to have flaccidity to the entire left side and decided to request air medical transport to minimize transport time to definitive care. The patient was last seen well by family an estimated eight hours before the arrival of EMS. Past medical history was only notable for hypertension, diabetes, and toxic multinodular goiter status post thyroidectomy. 

An air ambulance crew consisting of a prehospital physician, certified flight paramedic, and nurse orientee rendezvoused at an improvised landing zone near the patient’s residence. On arrival of the flight crew, the patient was found to have a patent airway with nonlabored respirations, a dense left hemiplegia with expressive aphasia, and a Glasgow Coma Scale score of 7 (E2, V1, M4). The right upper and lower extremities would withdraw to pain with full strength. The patient was also noted to be in a third-degree complete heart block with a heart rate (HR) of 48, blood pressure of 94/57 mmHg, end-tidal CO_2_ of 35 mmHg, with a pulse oximeter reading of 97% on 2 liters nasal cannula. The patient had several episodes of non-sustained ventricular tachycardia of a maximum of 10 beats. Glucose was confirmed to be 127mg/dL.

The initial clinical concern based on patient assessment was for ischemic versus hemorrhagic stroke. The Rapid Arterial oCclusion Evaluation (RACE) scale was completed and found to be suggestive of LVO; therefore, transportation to the closest comprehensive stroke center (CSC) was initiated per protocol. Given that the patient’s airway was patent without signs of compromise, and respirations were nonlabored, further airway management was deferred with plans to monitor closely for signs of deterioration. Despite the persistent third-degree heart block, the patient had an adequate mean arterial pressure (MAP) (>65 mmHg), peripheral capillary refill of less than two seconds, and bounding radial and dorsalis pedis pulses bilaterally. It was therefore surmised that the heart block was likely not the etiology of the patient’s altered mental status, and further interventions such as dopamine, epinephrine infusion, and transcutaneous cardiac pacing were considered but ultimately deferred in favor of minimizing time to definitive care. Aortic dissection was considered; however, due to symmetric pulses, it was felt to be less likely than a primary neurologic event.

**Figure 1:**
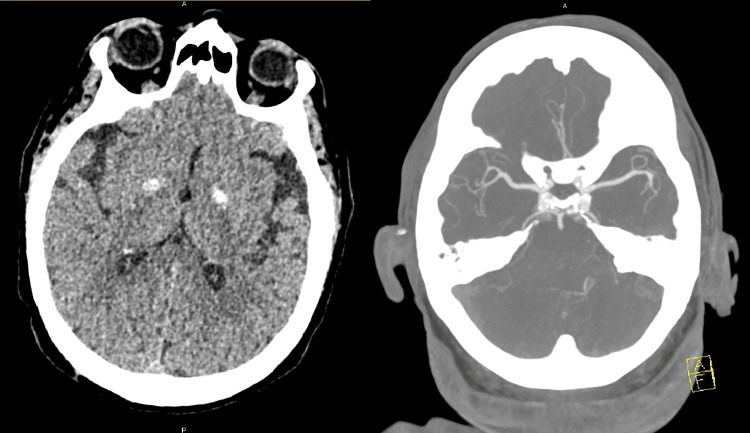
Non-contrast head CT and CT angiography findings Non-contrast head CT demonstrating bilateral basal ganglia dystrophic calcifications and absence of ischemic changes (left), and CT angiography demonstrating no large vessel occlusion (right) CT: computed tomography

On arrival at the hospital, the patient was noted to have spontaneous but weak motor activity in the left arm and leg. Upon arrival at the CSC, the patient was rapidly assessed by the emergency department and stroke neurology teams. A non-contrast head CT and CT angiography of the head and neck vasculature did not show acute findings, but incidentally revealed moderate to severe narrowing of the left PICA and dystrophic calcification of bilateral basal ganglia (Figure 1).

**Figure 2:**
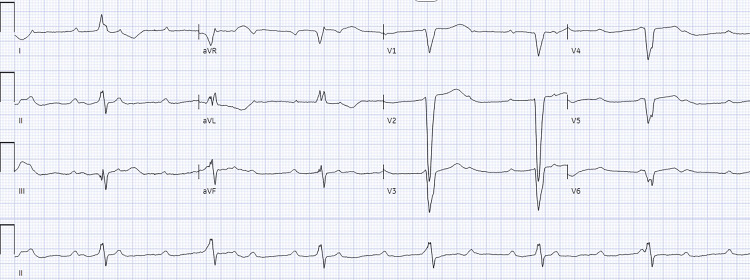
Electrocardiogram (ECG) demonstrating complete heart block

The patient was determined to be ineligible to receive intravenous thrombolytic (IVT) therapy due to the prolonged time from the last known well, and was not a candidate for mechanical thrombectomy given the absence of LVO. The patient remained in a complete heart block (Figure 2) with a heart rate in the 40s, MAP >65 mmHg, and no clinical signs of hypoperfusion. 

Laboratory values from initial ED testing are displayed in Table 1.

**Table 1:** Laboratory values BUN: blood urea nitrogen; PTH: parathyroid hormone; TSH: thyroid-stimulating hormone

Parameter	Patient value	Reference range
Sodium	141	136-145 mmol/L
Potassium	2.5	3.5-5.2 mmol/L
Chloride	120	98-107 mmol/L
CO_2_	10	22-30 mmol/L
Anion Gap	11	7-16 mmol/L
BUN	14	8-23 mg/dL
Creatinine	0.3	0.5-0.9 mg/dL
Glucose	93	70-99 mg/dL
Calcium	4.9	8.4-10.3 mg/dL
Lactic acid	1.8	0.5-2.0 mmol/L
PTH	4.7	11-68 pg/mL
TSH	6.83	0.4-4.0 mcIU/mL
Free T4	1.72	0.7-1.9 ng/dL

In the emergency department, the patient received 2 g of calcium gluconate as an intravenous infusion, as well as 40 mEq of potassium. Shortly after this, she was noted to have improvement in mental status, including answering orientation questions correctly and moving all four extremities spontaneously and with full and symmetric strength. The patient was ultimately admitted to the ICU, where she later underwent pacemaker placement due to persistent third-degree heart block in the setting of nonischemic cardiomyopathy. Echocardiogram demonstrated a left ventricular ejection fraction of 10-14%, and left heart catheterization showed no obstructive coronary artery disease. Her mental status and hemodynamics remained stable until she was discharged home in good condition several days later.

## Discussion

Stroke mimics are clinical conditions that present as symptoms or exam findings consistent with acute cerebral ischemia without an underlying ischemic or hemorrhagic process. Myriad pathologic disorders can mimic acute stroke, including migraine, peripheral vertigo, toxic/metabolic disturbances, psychiatric conditions, neoplasm, seizures, and functional disorders. The incidence of stroke mimic in acute neurologic presentations has been reported to range from 24.8 to 38% [4-6]. A study from a single helicopter EMS agency found that 20% of potential stroke incidents were mimics [7]. Stroke mimics are more commonly found in younger, female patients and are more likely to have a gradual onset rather than an acute one. One review found that patients were more aware of their surroundings and tended to track the examiner with their eyes, gaze deviation was uncommon, and exam findings were not anatomically distinct [8]. A review of the Get With the Guidelines-Stroke Registry found that 3.5% of all thrombolytic treatments were given to stroke mimics, although the rate of symptomatic intracranial hemorrhage was lower in mimics (0.4%) compared with ischemic stroke (3.5%) [9].

One comprehensive review found metabolic disturbances to be the second most common etiology of stroke mimic [4]. Severe hyponatremia can present with a variety of neurologic manifestations, including confusion, altered mental status, and seizures. Focal neurological symptoms, including hemiplegia, have been described in up to 18% of severely hyponatremic patients [10]. Hypoglycemia is another common electrolyte abnormality that may present as confusion, generalized weakness, as well as focal neurologic deficits. Hypokalemic paralysis (HP) is a neuromuscular disorder in which hypokalemia can manifest as symmetric proximal extremity muscle weakness. This can be periodic, as HP can result from an intracellular shift of potassium, as well as non-periodic, resulting from an overall large potassium deficit. While HP generally presents as symmetric muscle weakness, there are rare case reports of unilateral limb weakness [11]. While the mechanism of unilateral muscle weakness caused by hypokalemia remains unclear, some studies indicate that sodium-potassium pump activity may be asymmetrically distributed and vary in response to stress in different skeletal muscles. Recognition and prompt potassium replacement in these rare cases demonstrated an average recovery time of several hours to 48 hours.

Hypocalcemia in this case was likely caused by hypoparathyroidism due to the patient’s previous thyroidectomy, which may have inadvertently removed the parathyroid glands. Although the patient was on home calcium, vitamin D, and levothyroxine, it is unclear if she had been compliant with her medications. Hypocalcemia typically causes neuromuscular symptoms such as paresthesia, muscle cramping or weakness, or seizure. It also may cause hypotension, bradycardia, and heart block. There are rare case reports describing hypocalemia presenting as a transient ischemic attack, thought to be due to calcinosis of the basal ganglia and vascular structures [12]. Hypoparathyroidism has been suggested to cause vascular endothelial dysfunction, accelerated atherosclerosis, and vascular calcification, and has been associated with large vessel stroke in a pediatric patient [13]. Thyrotoxic periodic paralysis has been described to present with unilateral weakness and hypokalemia; however, this occurs in the setting of hyperthyroidism and has classically been described in men of Asian origin [14]. Conversely, hypothyroidism may present as confusion, generalized weakness, or altered cognition that might masquerade as a stroke. In this case, it was felt that the hypokalemia rather than hypocalcemia was likely the source of the patient’s neurologic symptoms.

Patients with insufficient cerebral perfusion in the setting of high-degree heart block and/or cardiogenic shock may present as a stroke mimic. In this case, the patient had no clinical signs of hypoperfusion in the prehospital setting or emergency department, and it was deduced that this was likely not the underlying factor contributing to her neurologic deficits. Todd’s paralysis due to seizure was also considered as a possibility, although the patient had no history of epilepsy or report of seizure activity from the husband. There were also no exam findings consistent with a seizure, such as tongue injury or incontinence. The rapid improvement in clinical examination following electrolyte replacement also suggests against this etiology. After a neurology consultation, it was ultimately felt by the patient’s primary care team that the significant electrolyte disturbance had been responsible for her initial neurologic presentation. However, some degree of diagnostic uncertainty remains in this case.

## Conclusions

We described a case of severe electrolyte abnormality-induced hemiplegia and altered mental status masquerading as an acute LVO stroke. Prehospital and emergency department clinicians should have a familiarity with and maintain a high index of suspicion for common stroke mimics.
